# Heme Oxygenase-1 as a Pharmacological Target for Host-Directed Therapy to Limit Tuberculosis Associated Immunopathology

**DOI:** 10.3390/antiox10020177

**Published:** 2021-01-26

**Authors:** Krishna C. Chinta, Hayden T. Pacl, Anupam Agarwal, Adrie J. C. Steyn

**Affiliations:** 1 Department of Microbiology, School of Medicine, The University of Alabama at Birmingham, Birmingham, AL 35294, USA; chait@uab.edu (K.C.C.); hpacl@uab.edu (H.T.P.); 2 Division of Nephrology, School of Medicine, The University of Alabama at Birmingham, Birmingham, AL 35294, USA; aagarwal@uabmc.edu; 3 Birmingham Veterans Affairs Medical Center, Birmingham, AL 35294, USA; 4 Africa Health Research Institute, University of KwaZulu-Natal, Durban 4001, South Africa; 5 UAB Centers for AIDS Research and Free Radical Biology, The University of Alabama at Birmingham, Birmingham, AL 35294, USA

**Keywords:** heme oxygenase-1, *Mycobacterium tuberculosis*, TB, macrophages, neutrophils, reactive oxygen species, reactive nitrogen species, immunopathology, immunometabolism, glycolysis, pentose phosphate pathway

## Abstract

Excessive inflammation and tissue damage are pathological hallmarks of chronic pulmonary tuberculosis (TB). Despite decades of research, host regulation of these clinical consequences is poorly understood. A sustained effort has been made to understand the contribution of heme oxygenase-1 (HO-1) to this process. HO-1 is an essential cytoprotective enzyme in the host that controls inflammation and oxidative stress in many pathological conditions. While HO-1 levels are upregulated in animals and patients infected with *Mycobacterium tuberculosis* (*Mtb*), how it regulates host responses and disease pathology during TB remains unclear. This lack of clarity is due in part to contradictory studies arguing that HO-1 induction contributes to both host resistance as well as disease progression. In this review, we discuss these conflicting studies and the role of HO-1 in modulating myeloid cell functions during *Mtb* disease progression. We argue that HO-1 is a promising target for host-directed therapy to improve TB immunopathology.

## 1. Introduction

*Mycobacterium tuberculosis* (*Mtb*), the causative agent of tuberculosis (TB), has coevolved with humanity for as many as 3 million years [1]. An enormously successful pathogen, *Mtb* caused estimated 10 million new TB cases and 1.2 million deaths globally in 2019 alone [2]. Though an effective regimen exists for TB, its length and complexity results in low adherence in patients [3]. Complicating this issue, inconsistent treatment has contributed to the emergence of multidrug resistant and extensively drug-resistant strains of *Mtb* and, ultimately, to the persistence of the pathogen [2,3]. Finally, the TB epidemic is also exacerbated by comorbidities such as human immunodeficiency virus (HIV) and diabetes which have further contributed to the global mortality caused by *Mtb* [4]. 

**Figure 1:**
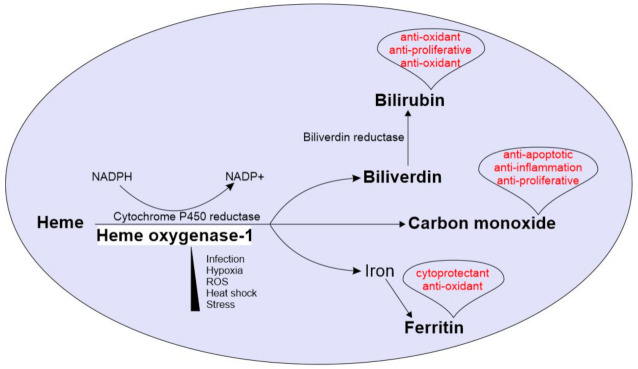
Overview of Heme oxygenase-1 (HO-1) enzymatic reaction and physiological roles of its enzymatic by-products: HO-1 is induced under various physiological challenges including stress, hypoxia, ROS, heat shock and microbial infections. The HO-1 catabolizes heme into equimolar ratios of CO, iron and biliverdin, using NADPH and cytochrome p450 reductase. Biliverdin is further converted into bilirubin by biliverdin reductase. The free iron is stored by the iron storage enzyme ferritin. CO, bilirubin and ferritin drives several host protective physiological roles including anti-inflammation, cryoprotection and anti-oxidation. Physiological roles of each by-product are in red font.

As with many other pulmonary diseases, TB is characterized by extensive tissue damage driven by the immune response. The current anti-TB therapy predominantly targets bacterial replication and direct killing of the bacillus, but it does little to improve disease pathology. There is, therefore, tremendous interest in host-directed therapies (HDTs) aimed at either improving the antimicrobial response of host immune cells or restricting inflammation, thereby limiting tissue damage [5]. To this end, a promising candidate for improving TB immunopathology is the inducible antioxidant protein, heme oxygenase-1 (HO-1). HO-1 catabolizes the highly potent, pro-oxidant molecule, heme, into equimolar ratios of carbon monoxide (CO), iron, and biliverdin (BVD) [6]. In addition to removing the pro-oxidant heme, HO-1-derived CO and BVD serve anti-inflammatory functions. BVD can be converted to the antioxidant, bilirubin; together, BVD and bilirubin can function as an intracellular antioxidant against oxidative species [7,8]. Through more nuanced mechanisms, CO has been shown to be antiapoptotic, antiproliferative, and anti-inflammatory [9,10]. Lastly, although iron is also a pro-oxidant, it induces the expression of ferritin H, which sequesters free iron, thus protecting the cell from a potentially toxic product [11]. Further underscoring its importance, HO-1 deficiency is ultimately lethal in humans [12,13]. Therefore, HO-1 and its enzymatic activity protect the host from oxidative stress through several important pathways, and together with its inducible nature, HO-1 makes a compelling candidate for HDT to limit TB immunopathology (Figure 1). 

While the regulation of free heme has not been addressed explicitly in TB, it has been explored as a therapeutic approach for other diseases. In fact, the therapeutic administration of hemopexin (Hx), a molecular scavenger of heme, is known to rescue macrophages from heightened heme-mediated proinflammatory responses in a mouse model of sickle cell anemia [14]. In line with this, therapeutic manipulation to induce the levels of HO-1 or its enzymatic products has been suggested as a potential clinical intervention to protect normal lung architecture and limit disease progression during pulmonary diseases including chronic obstructive pulmonary disease, cystic fibrosis and asthma [15,16]. HO-1 mediated cytoprotection has also been observed during salmonellosis [17], renal diseases [18], malaria [19], and mycobacterial infections [20,21,22,23]. On the other hand, a few studies suggest that the induction of HO-1 has a detrimental effect on the host during infection with various pathogens, including *Mtb* [24,25,26]. However, many of the studies involving *Mtb* report associations, and the conclusions from those studies are not tested clinically. For example, *Mtb* infection increases HO-1 expression in macrophages cultured in vitro and in the lungs of *Mtb* infected mice. Additionally, elevated levels of plasma HO-1 have been found in patients with active pulmonary and extrapulmonary TB and is suggested as a potential diagnostic marker for patients with active pulmonary and extrapulmonary TB [27]. Due to the lack of experimental controls, however, the role of HO-1 in the host response to *Mtb* infection and disease progression remains elusive.

Lastly, while the precise mechanism of HO activity during TB, especially in humans, is not clearly defined, it is well known that hemoptysis and pulmonary hemorrhage are some of the best known clinical hallmarks of human TB [28]. Hence, elevated levels of heme from hemoglobin may overwhelm the cytoprotective functions of HO-1 and contribute to the immunopathology of TB by dysregulating oxidative, inflammatory, and iron homeostasis [20,28]. Beyond simply inducing oxidative stress, heme, whether from hemorrhage or not, may be a central driver of the dysregulated myeloid cell response and subsequent tissue destruction observed during chronic TB. For example, heme generates reactive oxygen species (ROS) which promotes lipid peroxidation and DNA damage in macrophages, a major cell type involved in the host response to *Mtb* infection [29,30]. Similar observations were found in our recently published study where the macrophages and neutrophils isolated from severely damaged regions of resected human TB lungs had low expression of HO-1 and higher ROS and reactive nitrogen species (RNS) levels compared to healthy regions suggesting a cytoprotective role for HO-1 [20]. 

## 2. Scope

In this review, we critically analyze the studies arguing that HO-1 induction improves immunopathology in TB along with the studies that suggests it is deleterious to the host. We reconcile their key findings and discuss previously unanswered questions pertaining to the role of HO-1 in TB disease. We focus primarily on the role of HO-1 in modulating macrophage and neutrophil responses during TB and contextualize it within the concept of the immunometabolism. We argue that HO-1 is a compelling target for HDT given its potential to improve energy homeostasis in these cells. Lastly, we discuss HO-1 deficient mouse models of TB and the discrepancies surrounding the benefits or consequences of HO-1 deficiency in humans and mice, which has not received much attention in most of the TB HO-1 studies to date.

## 3. Myeloid Cells in *Mtb* Immunopathogenesis

The initial infection in TB occurs when *Mtb* infects alveolar macrophages (AMs), which phagocytose, but fail to kill *Mtb* [31]. Infected AMs then secrete chemokines and recruit macrophages, neutrophils, natural killer cells, and dendritic cells, leading to the induction of adaptive immunity. Ultimately, infiltrating immune cells form an organized cellular architecture called the necrotic granuloma, the histological hallmark of TB [32]. Of these immune cells, macrophages and neutrophils play a major role in the progression of TB, as they are the primary reservoir for *Mtb* in vivo and are suggested to both protect against and contribute to TB pathogenesis [33,34]. For instance, one of the major macrophage and neutrophil responses against *Mtb* is generation of ROS and RNS, respectively, such as superoxide, hydrogen peroxide, hypochlorite, nitric oxide (NO), and peroxynitrite, as well as antimicrobial peptides such as human neutrophil peptide-1 [35]. However, failure to regulate these responses may contribute to disease pathology via uncontrolled cell death, extensive tissue damage, and ultimately, a complete loss of lung function in the affected areas of the lung [20,36].

The primary function of macrophages in alveolar spaces and within the granuloma is to restrict *Mtb* dissemination and promote bacterial killing. However, recent studies have shown that the macrophage ontology and heterogeneity play critical yet diverse roles during *Mtb* infections. In a mouse model of *Mtb* infection, AMs have been shown to provide a pathogen permissive environment whereas interstitial macrophages restrict *Mtb* growth [37]. Similarly, macrophage heterogeneity and polarization have been suggested to govern granuloma formation, either to build host resistance against *Mtb* via early inflammatory responses such as NO and cytokine release, or providing a niche for bacteria and contributing to immunopathology by creating immune suppressive environment [38,39,40]. Overall, this conundrum establishes a biphasic paradigm for the optimal immune response against *Mtb*: inflammation is beneficial during the early, but not late stages of disease. With this in mind, it is clear that the ontology, activation state, and phenotype of macrophages are essential in determining the outcome of *Mtb* infection. Furthermore, metabolic state of the macrophages is a critical determinant of its activation and function and therefore the nutrient availability within the TB granuloma microenvironment may also determine the outcome of the infection [41,42]. Ultimately, the plasticity of macrophage phenotype and its role in TB pathogenesis make macrophages a compelling target for HDT. 

Like macrophages, neutrophils also participate in TB pathogenesis, albeit with fewer redeeming qualities. There are conflicting data surrounding whether neutrophils are capable of killing *Mtb* [43], how neutrophils undergo cell death after *Mtb* infection [44,45], and if they play a helpful or harmful role in TB [46,47]. They are tied directly to disease in humans by studies showing that neutrophils are the most abundant phagocyte in sputum samples, in the airways of TB patients, and among cells infected with *Mtb* [48], as well as being associated with human pulmonary TB cavities [20,49]. Therefore, as with macrophages, neutrophils may be protective early in *Mtb* infection, but they seem to be ineffective in controlling *Mtb* infection, or, worse, a major contributor to TB pathology. There is increasing evidence that there is a massive influx of neutrophils during the chronic stages of infection in both animal models and in TB patients [43,48,50]. Lastly, neutrophil numbers, their proportions compared to other lymphocytes and natural killer cells, and neutrophil specific transcriptional profiles have been proposed as diagnostic and prognostic indicators in human TB [51,52]. Given that neutrophils contribute to disease, persist in the tissue during active disease, and contribute in a limited manner to the control of *Mtb*, neutrophils also comprise a compelling target for HDTs in TB [53,54].

## 4. Challenges in Studying the Role of HO-1 in Mice versus Humans

As discussed in the introduction, one of the major goals of identifying new HDTs is to limit inflammation-mediated tissue damage in TB. However, most studies aimed at identifying promising HDTs use animal models, especially mice and lack translation to humans. To improve the translation of findings into treatments, it is crucial to address the fundamental differences between humans and experimental mouse models with regard to major immune functions. Specifically, there are significant differences in the expression of antimicrobial peptides, including defensins, toll receptors, iNOS, and HO-1 [55,56]. Directly relevant to this review, there is a significant difference in the requirement for HO-1 among humans and mice. While HO-1 deficiency in mice is often lethal at the prenatal stage, mice that survive this stage have a lifespan comparable to wild-type animals [6]. In contrast, HO-1 deficiency in humans is lethal [12,13]. In both known cases, HO-1 deficient patients showed uncontrolled systemic inflammation, hemolysis, hemorrhage, and elevated levels of extracellular heme leading to early death. Interestingly, this description matches the clinical hallmarks of active TB patients who progress to require surgical lung resection [20]. 

In addition, the molecular regulation of the human HO-1 gene differs from the regulation of the mouse HO-1 gene [57]. It is important to study HO-1, or any other proposed targets for HDT for that matter, within the context of inter and intralesion heterogeneity in TB. As mentioned previously, the granuloma is a dynamic microenvironment that undergoes changes in composition and cellular architecture, and these changes can ultimately impact clinical outcomes [58]. This is especially important in the context of understanding the contribution of myeloid cells, as their mere presence is not enough to understand their contribution to host protection or harm. For example, in a recent study, we measured the expression of HO-1 within macrophages and neutrophils isolated from pathologically distinct regions of resected human TB lung and showed that the regions of severe damage have the lowest HO-1 expression [20]. Interestingly, the myeloid cells from these severely damaged regions have significantly higher levels of ROS and RNS, suggesting that HO-1 regulates redox homeostasis and protects against immunopathology in human TB [20]. While the convenience of the mouse model of TB certainly justifies its use, we argue that it is essential to complement such experiments with studies that contextualize findings as seen in human TB. Routine access to fixed and, preferably, freshly resected lung tissue is essential for understanding the contribution of immune and histological heterogeneity. However, we acknowledge that these resources are scarce. BAL fluid and blood from patients are reasonable stop-gap measures for assessing cumulative readouts of the complex systemic response to TB, but their value is largely limited to describing correlates of disease and does not provide any information on the immune architecture of the lung microenvironment which is the primary site of infection. 

## 5. HO-1 as a Diagnostic or Prognostic Indicator

As with many diseases, the challenge of treating TB begins with diagnosing it. Currently, the most used diagnostic methods include the sputum smear test to microscopically detect acid-fast bacilli, the tuberculin skin test (TST), the IFNγ-release assay (IGRA), and radiological imaging. However, these various approaches have significant limitations including their accuracy and their ability to distinguish between latent and active TB cases [59,60,61]. It is not surprising, then, that there has been substantial interest in various biomarkers, such as HO-1, as indicators of TB disease states. HO-1 levels in plasma, for example, were found to be significantly higher in patients with active pulmonary or extrapulmonary TB compared to latently infected individuals, distinguishing latently infected individuals from actively infected TB patients [27]. In fact, HO-1 levels in individuals with latent tuberculosis infection were comparable to healthy controls, thus clearly distinguishing them with the active TB patients [27]. Interestingly, following successful drug treatment, the authors observed a reduction in the plasma HO-1 level, which was also comparable to the healthy controls. While other proteins, such as serum amyloid A protein and C-reactive protein have been proposed as biomarkers to distinguish latent and active TB patients [62], HO-1 served as a better marker to distinguish between latent and active pulmonary and extrapulmonary TB patients [27]. Further, the levels of HO-1 in plasma positively correlated with IL-10 and negatively with TNFα, suggesting that the elevated levels of HO-1 maybe involved in regulating the inflammatory responses during active pulmonary TB [27]. Similarly, circulating HO-1 was also suggested as a promising biomarker in detecting pulmonary and extrapulmonary TB in children [63,64]. Plasma HO-1 levels were also elevated in TB-patients with type-2 diabetes mellitus, independent from *Mtb* burden. These increased plasma HO-1 levels positively correlated with IFNγ, TNFα, and interleukin-17A, tissue inhibitors of metalloproteinase-4, and blood neutrophil numbers [65]. Interestingly, HO-1 in the plasma is also positively correlated with plasma glucose, glycosylated hemoglobin, and low-density lipoprotein levels, suggesting a potential role for HO-1 in modulating energy requirements [65]. In a more recent study by Rockwood et al., elevated levels of plasma HO-1 were also observed in TB/HIV coinfected patients [66]. Consistent with previous findings, increased HO-1 levels were higher in the patients who did not receive anti-TB therapy and in those whose therapy failed [66]. The authors suggested that this increase in plasma HO-1 levels in TB patients is dependent on *Mtb* early-secreted antigen 6-induced NADPH oxidase-mediated ROS production [66]. Lastly, the HO-1 polymorphisms are also suggested to be indicative of TB susceptibility and anti-TB drug-induced liver injury [67,68]. In a controlled study consisting of over 600 pulmonary and extrapulmonary TB patients and healthy controls, Wu et al. showed a positive correlation between single nucleotide polymorphisms in HO-1 and the susceptibility to TB [67]. Similarly, the genetic polymorphisms in HMOX1 were associated with anti-TB drug-induced liver injury in Chinese demographics [68]. 

Altogether, these studies make a strong case for the use of HO-1 as a diagnostic or prognostic marker, especially in extrapulmonary and sputum-smear negative cases. However, further interpretation should be made cautiously. Firstly, there is little mechanistic explanation for how HO-1 accumulates in the plasma, given that HO-1 is generally considered to be an intracellular protein. Elevated levels of HO-1 are observed in several other pulmonary diseases such as acute respiratory distress syndrome [69], chronic obstructive pulmonary disease [70], cystic fibrosis [71], and asthma [72]. Like TB, these pathological conditions often involve exaggerated inflammation resulting in of the disruption of normal lung parenchyma. However, unlike the studies in the blood of TB patients, HO-1 levels in these respiratory diseases were measured in lung tissues and BAL fluids which provides a better representation of the disease microenvironment. In an acute kidney injury (AKI) study, Zager et al. showed that plasma and urinary HO-1 levels are increased in mice and in clinical samples [73]. The authors hypothesized that tubular damage to the plasma membrane could result in the release of renal HO-1 into the circulation. In addition, damage to other cellular compartments such as mitochondria and endoplasmic reticulum could also result in elevated plasma or urine HO-1 [73]. Given that acute or chronic TB results in significant loss of lung tissue, HO-1 originating from cells within the lung may significantly contribute to elevated plasma HO-1 levels [20]. 

Secondly, the elevated levels of plasma HO-1 do not provide any information about HO-1 levels within the lungs, the primary site for *Mtb* infection, or if the differences seen between active and latent TB could also be present at the site of infection. Interestingly, HO-1 levels in neutrophils isolated from TB patients are significantly lower than those in healthy individuals, further highlighting the fact that the levels observed in plasma do not directly relate to HO-1 expression in the lungs [20]. With regards to the first issue, further studies are required to increase our knowledge of the microanatomical distribution of HO-1 within the lungs of TB infected patients to better understand if HO-1 production at the site of disease contributes to serum HO-1 levels. Lastly, underlying these findings, the detection of plasma HO-1 levels in TB patients was done by ELISA, which does not exclusively detect the functional HO-1 enzyme. For example, Zager et al. showed that HO-1 detected in the plasma and urine was a truncated 16-kd fragment of protein (full-length HO-1 has a molecular weight of ≈34.56 kDa) and therefore could potentially be enzymatically inactive [73]. While this does not directly impact the value of this test as a biomarker, it impacts our ability to infer the role of this protein in the pathogenesis of TB. Taken together, further investigation is required to establish the relevance and functionality of elevated plasma HO-1 levels and *Mtb* disease progression.

## 6. The Controversy: Is HO-1 Host Protective or Harmful during *Mtb* Pathogenesis?

Upregulation of HO-1 production has clearly been observed upon *Mtb* infection, in vitro in macrophages, in vivo in mice, and in the plasma of active TB patients [20,27,74]. The upregulation of HO-1 was initially an exciting finding in the context of work from Kumar et al., who demonstrated that the DosR/S/T three-component system in *Mtb* can sense CO and is responsive to CO [74,75]. HO-1-derived CO, therefore, can induce the expression of the Dos dormancy regulon in *Mtb* [74,76]. As with much of the HO-1 literature in the TB field, it is unclear whether the induction of the Dos dormancy regulon ultimately protects the host or the pathogen. However, in a macaque model for TB, infection with an *Mtb* dosS mutant, the animals were able to clear the infection, suggesting that induction of the Dos dormancy regulon via DosS is important for successful infection [77]. Therefore, it can be argued that HO-1/CO-mediated induction of the Dos dormancy regulon promotes disease. The matter is further complicated, however, by a poor understanding of the impact of an increase in intracellular free iron following heme degradation by HO-1 on *Mtb* virulence. Lastly, the overall impact of HO-1 in countering oxidative stress and inflammation, the control of *Mtb* infection, and ultimately TB pathology is also strongly debated.

To this end, several recent studies suggest that inhibiting HO-1 may be beneficial for the host. In an in vitro macrophage model of TB, Scharn et al. show that chemical inhibition of HO-1 using tin protoporphyrin IX (SnPP) leads to a reduction in the secretion of proinflammatory cytokines, including IL-1β, TNF-α, and IL-6. Additionally, they showed that HO-1 enzymatic activity increases iron availability within macrophages, which the authors suggest facilitates *Mtb* growth. Similarly, several reports have demonstrated that a lack of HO-1 increases iron availability in macrophages and contributes to iron-mediated oxidative stress and tissue damage [6,78]. However, HO-1 induction after *Mtb* infection also induces the levels of Ferritin-H (Fth) which is required for host protection against *Mtb* infection [28]. Of importance, Reddy et al. showed that Fth stores free iron in insoluble, metabolically inert aggregates known as hemosiderin, which is inaccessible to both host and the bacteria [28]. Using iron van Gieson stain of human TB lungs, Reddy et al. showed that the granulomatous regions predominantly expressed hemosiderin and suggested that there is no free iron available in the TB lungs that can be used by *Mtb* for its growth [28]. Overall, it is unlikely that HO-1 enzymatic activity results in increased iron availability for *Mtb*. Furthermore, the papers demonstrating a survival benefit for *Mtb* following the induction of HO-1 were demonstrated in vitro and not in vivo. This limits the broader impact of HO-1-mediated host benefits, such as reducing tissue damage, lowering bacterial burden, and increasing survival, as observed in our study [20]. In this study, two distinct HO-1 deficient mouse models were used; in the one model system, HO-1 was globally knocked out in all cell types and in the second LysM-Cre model, only myeloid cells were HO-1 deficient. Notably, we observed that both HO-1 deficient mouse models were highly susceptible to *Mtb* with elevated inflammatory markers and increased disease pathology [20]. 

There is some in vivo evidence that supports inhibition of HO-1 as a therapeutic approach for TB. Costa et al. showed that treating mice with SnPP reduced the bacterial burden of mice upon *Mtb* infection by approximately 10-fold [25]. However, the relatively shorter duration for post infection bacterial counts and lack of longer timepoints limits broader generalization, since TB is a chronic disease and longer time points are needed to establish the effect of treatment on disease outcome. Although, the authors posit that pharmacological inhibition of HO-1 plays a protective role by regulating T-cell responses, the protective effect observed in *Mtb* infected WT mice treated with SnPP was absent in a transgenic TCRα KO mouse. However, there is considerable debate regarding the use of SnPP to inhibit HO-1 enzymatic activity, and SnPP has significant side effects and toxicity, including significant erythema, hemolysis, and elevated levels of ROS leading to increased mortality in rats and guinea pigs [79,80,81,82]. Additionally, the survival data showed no specific effect of SnPP treatment and the survival was only compromised upon the deficiency of TCRα [25]. These results emphasize the importance of TCRα-/- during *Mtb* infection as was shown by others [83,84]. However, the lack of mechanistic explanation of SnPP mediated regulation of T-cell responses clearly undermines these findings. Notably, there is the danger that inhibiting HO-1 could lead to elevated levels of heme followed by massive inflammation, and tissue damage via ROS and lipid peroxidation [20,29,30]. Ultimately, however, there is the likelihood that HO-1 may have a different role during *Mtb* pathogenesis, where its upregulation during the early and late stages have opposite effects. During the early stages of infection, the anti-inflammatory activity of HO-1 may limit the early innate immune responses and thereby contributing to the bacterial survival. This was suggested in a macrophage model of Salmonella, where HO-1 upregulation restricted the early innate immune responses thereby contributing to disease progression [17]. Consistent with this, in another study, the authors suggested that reduction in *M. abscessus* bacterial burden in SnPP-treated macrophages could be due to the restriction of early host innate immune responses [24]. However, inhibiting HO-1 could be deleterious to the host, especially during active TB when there is an abundance of heme due to massive hemorrhage as observed in human TB patients [20,28]. 

Taken together, for the reasons given above, it is reasonable to suggest that pharmacological inhibition of HO-1 should be avoided as an HDT for TB patients. This complexity also emphasizes the importance of correlating findings observed in animal models with studies in humans. In one such study, Chinta et al. demonstrated using freshly resected human TB lung tissues to study the inflammatory responses in pathologically distinct regions that myeloid HO-1 levels are important to limit TB pathology [20]. Importantly, these findings were coupled with experiments in two independent mouse models showing that the deficiency of HO-1 either globally or specifically within the myeloid cells were more susceptible to *Mtb* infection [20]. Additionally, the study showed that HO-1 levels in the wild type mice decrease at late stages of infection (40 weeks), which demonstrates that HO-1 deficiency coincides with mortality during the later stages of *Mtb* infection [20]. 

## 7. HO-1 as a Regulator of Myeloid-Cell Immunometabolism in TB

Immunometabolism is a reemerging field of study which explores the intersection of metabolism and function in immune cells [85]. Major metabolic pathways including glycolysis, pentose phosphate pathway (PPP), tricarboxylic acid cycle, oxidative phosphorylation, fatty acid synthesis, and fatty acid oxidation have been shown to significantly modulate the effector function of immune cells [86]. Specifically, in recent years immunometabolism in TB has gathered attention, and recent studies have explored the dynamic changes in the metabolic pathways of immune cells upon *Mtb* infection [87,88]. These studies showed that *Mtb* infection induces a metabolically quiescent state and limits the anti-*Mtb* responses in both, in vitro macrophages and T-cells isolated from *Mtb*-infected mice [87,88]. Fitting into this novel paradigm, HO-1 has long been appreciated for its role in modulating metabolism [89] and may play a role as a key metabolic regulator in myeloid cells during TB.

### 7.1. Targeting HO-1 Expression in Macrophages to Limit TB Immunopathology

Macrophage functions are critical for host protection against *Mtb* disease progression. In this regard, the immunometabolism of macrophages plays a crucial role in modulating the host response during TB and provides many potential therapeutic avenues to limit disease progression. In a recent study, Cummings et al. showed that *Mtb* infection leads to quiescent bioenergetics phenotype macrophages [87]. Using extracellular flux analysis, the authors showed that there are significant differences in the mitochondrial respiratory profile of macrophages infected with virulent *Mtb* compared to macrophages infected with BCG or dead *Mtb*. Virulent *Mtb* infection significantly reduces the spare respiratory capacity and maximal respiration compared in hMDMs [87]. Interestingly, upon BCG infection, hMDMs showed increased spare respiratory capacity and maximal respiration [87]. Spare respiratory capacity is important for macrophage adaptation to stressors in the surrounding microenvironment, including changes in nutrient availability, redox imbalances, and pH fluctuations, among other factors. Therefore, therapeutic manipulation directed towards improving the spare respiratory capacity of macrophages could be beneficial to the host. To this end, HO-1 induction could be exploited as it was shown to rescue the respiratory profiles in lung fibroblasts from chronic obstructive pulmonary disease patients [90]. Similarly, exogenous CO treatment using carbon monoxide-releasing molecule-401, which releases a controlled amount of CO, has been shown to significantly increase the maximal respiration and was suggested as a treatment to improve endothelial cell-related pathologies [91].

Another interesting approach to modulating macrophage functions could be by targeting the macrophage phenotype, ranging between proinflammatory (M1) and anti-inflammatory (M2) phenotype, by targeting macrophage metabolism [92,93]. Within the TB granuloma, the predominant phenotype of macrophage population is a key determinant of disease progression [40]. Using a computational-biology approach, Marino et al. suggested that pharmacologically promoting the M1 phenotype, especially during early stages of infection could improve the disease outcome [40]. In contrast, it is important to mention that regulating the proinflammatory responses is crucial to limit inflammation-mediated pathology [94]. The latter is especially important because the clinical hallmarks of chronic TB include massive tissue damage, as discussed earlier. In this regard, HO-1 expression has been linked to the regulatory macrophage phenotype [95,96]. In addition, it is also important to note that metabolic changes which in turn can be regulated by HO-1, can also influence the phenotype changes in macrophages. M1 macrophages largely depend on glycolysis to carry out their proinflammatory responses [97,98]. Conversely, M2 macrophages rely on oxidative phosphorylation and glycolysis is largely dispensable in these cells [99]. The role of CO in modulating these pathways has been investigated in brain and cancer cells where exogenous CO exposure resulted in increased OCR, decreased glucose usage, and decreased lactate production [100,101]. These studies provide a gateway to study the role of HO-1 in modulating macrophage metabolism that ultimately results in macrophage shift towards M2 phenotype. In sum, it is reasonable to postulate that maintaining consistent levels of HO-1 or CO within macrophages will limit the proinflammatory responses, the avenue that can be targeted to limit proinflammation mediated damage during TB. 

Lastly, modulating macrophage MMP secretion by targeting HO-1 represents yet another approach to limit TB pathology [65]. MMPs are major contributors to host immunopathology during active TB disease [102,103]. Similarly, as discussed in Section 4, MMP-1 levels are high in patients with TB, while the levels of TIMP were significantly lower. Further, using transgenic mice expressing human MMP-1, it was demonstrated that MMP-1 expression results in significant loss of normal lung parenchyma [102]. In this regard, HO-1 has been shown to reduce MMP levels significantly via inhibition of IL-1β pathway which is required for the production of MMPs [104]. Similarly, the pharmacological induction of HO-1 has been shown to significantly reduce the MMP1 levels in *Mtb*-infected macrophages. In a recent study, it was shown that the levels of both HO-1 and MMP-1 are significantly elevated in the plasma of active TB patients compared to healthy controls and individuals with latent tuberculosis infection [65]. Interestingly, in a similar study by Andrade et al. that examined a diverse cohort, plasma levels of HO-1 inversely correlated with MMP-1 in patients with active TB and had distinct inflammatory biomarker profiles [105]. As mentioned earlier, the authors also found that infection of macrophages with *Mtb* lead to induction of HO-1 and not MMP-1 [105]. Moreover, exposure of *Mtb* infected macrophages to a HO-1 inhibitor SnPP significantly elevated MMP-1 expression. Conversely, exposure to cobalt protoporphyrin (CoPPIX), a potent HO-1 inducer, decreased MMP-1 expression. Notably, HO-1-mediated regulation of MMP-1 in macrophages was due to the direct effect of CO via the suppression of c-JUN/AP-1 and not by other HO-1 enzymatic by-products. This further shows that pharmacological regulation of HO-1 as an HDT within the microenvironment of TB granuloma may potentially limit TB immunopathology. 

### 7.2. HO-1 Regulates Macrophage Function to Maintain an Organized Granuloma

Compelling evidence supporting a role for HO-1 in the host response during *Mtb* infection, comes from studies investigating a related pathogenic mycobacterial species: *Mycobacterium avium* (*Mav*). Similar to *Mtb*, *Mav* presents as a pulmonary infection and promotes granuloma formation within the host. To date, several studies identified HO-1 as an essential component in forming the granuloma in mice infected with *Mav* [21,22,106]. The first of these studies demonstrated that HO-1 is induced in the normal course of *Mav* infection and is observed within the granuloma in wild type mice, whereas HO-1-deficient mice fail to form organized granulomas [21]. In a follow-up study, this group suggested that the phenomenon of dysregulation in granuloma formation is responsible for the increased risk for Mav infections observed in the elderly—a population characterized by an impaired ability to induce the expression of HO-1 [107,108]. Regev and colleagues attributed the observed defect in granuloma formation to impaired immune cell recruitment at the granuloma, as they observed inhibition of MCP-1 and induction of CCR2, a key signaling axis in monocyte and macrophage trafficking in a murine cell line treated with the HO-1 inhibitor zinc protoporphyrin-IX [21]. Supporting this, GFP-labeled peripheral blood monocytes transferred to wild type mice localized within the granuloma, whereas when transferred to the HO-1 deficient mice, the transferred monocytes spread diffusely throughout the lung suggesting that HO-1 is required for the formation of organized granuloma in mice [21]. Of note, higher levels of MCP-1 were detected in the bronchoalveolar lavage fluid of HO-1 deficient mice relative to wild-type mice at baseline and after infection, which is consistent with increased systemic inflammation observed in HO-1 deficient mice [21].

**Figure 2:**
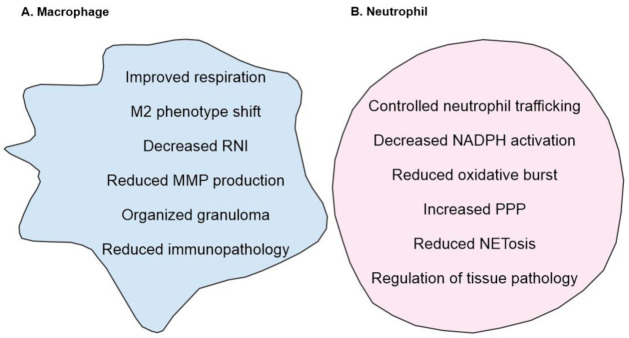
Overview of HO-1 mediated regulation of (**A**) macrophages, (**B**) neutrophils. (**A**) HO-1 improves mitochondrial respiration which in turn can modulate macrophage phenotype towards M2 phenotype. In addition, increase in HO-1 levels correlates with decreased MMP and RNI levels. (**B**) In neutrophils, HO-1 controls oxidative burst, decreases NADPH activation and shifts the neutrophil metabolism towards PPP. In addition, HO-1 also reduced the formation of NETosis and regulates neutrophil trafficking and recruitment. Together, these responses result in decreased inflammation and tissue pathology.

Poor granuloma formation and increased expression of CCR2 in HO-1 deficient mice infected with *Mav* were also observed by Silva-Gomes and colleagues, with the added observation that the protective role of HO-1 is independent of adaptive immunity. This group attributed their findings to the pro-oxidant nature of heme rather than a dysregulated MCP-1/CCR2 axis and suggested that losing the ability to degrade heme leads to a cytotoxic effect on infected macrophages, citing a prior study that showed no defect in the clearance of *Mtb* infection in MCP-1 deficient mice [109]. In the study cited by Silva-Gomes and colleagues, however, *Mtb* were administered intravenously; since the natural route of infection is via the respiratory system and one role of the granuloma is to prevent dissemination of mycobacteria, there are numerous possible explanations for the lack of difference in *Mtb* clearance in this model that may conceal an essential role for MCP-1/CCR2 in the granulomatous response to mycobacteria. Ultimately, however, these studies are not in conflict; rather, they both demonstrate a crucial role for HO-1 in granuloma formation secondary to mycobacterial infection (Figure 2).

### 7.3. Targeting HO-1 Production in Neutrophils to Limit TB Immunopathology

As with any therapy that intends to modify immunity, therapies targeting neutrophils must suppress function enough to have an impact, but suppression must be limited so as not to invite opportunistic infection. It is in this context that HO-1 is a target of particular interest. HO-1 regulates neutrophil functions such as trafficking and oxidative burst in a substantial, but controlled manner. Freitas et al. observed that HO-1 and its products BVD and CO reduce neutrophil rolling, adhesion, and migration in the context of sterile inflammation [110]. This effect was dose-dependent, and treatment with HO-1 inhibitors showed the opposite effect. Delving into potential mechanisms, the authors identified that CO dependent effects appear to be mediated through soluble guanylate cyclase, while HO-1 activity may mediate the inhibition of neutrophil trafficking observed after treatment with a nitric oxide donor [111,112]. Interestingly, these findings support the hypothesis that HO-1 maintains soluble guanylate cyclase in a reduced state capable of responding to NO [113]. Thus, there appears to be a role for HO-1 in modulating the trafficking of neutrophils to inflammatory sites. This bears particular relevance to TB, as excessive neutrophilic infiltration drives the pathology of TB [114].

Another way in which HO-1 may regulate neutrophil function is by modifying the oxidative burst. The induction of HO-1 decreases activation of the NADPH-oxidase components, p47phox and p67phox, in murine neutrophils [115]. This inhibited assembly of the complex and the production of superoxide results in decreased tissue damage in a model of post-burn injury. More recently, HO-1 induction in the bone marrow was shown to have a lasting impact on the ability to develop neutrophils to produce an oxidative burst [116]. For example, HO-1 inhibition for 48 h restored the oxidative capacity of developing neutrophils, but not mature neutrophils. This observation was made in a coinfection model where HO-1 was induced by infection with *Plasmodium yoelii* and the subsequent oxidative defect impaired resistance to non-typhoid Salmonella. While at first glance this suggests that the induction of HO-1 may not be a valuable therapeutic in a bacterial disease, the inability of neutrophils to kill *Mtb* in the context of neutrophil-mediated immunopathology suggest that neutrophil targeted immunosuppression may be beneficial in TB. Furthermore, the ability to restore the oxidative capacity in developing neutrophils within 48 h by inhibiting HO-1 provides a means to tailor a potential treatment or reverse the effect of this treatment relatively quickly.

Lastly, metabolic changes in neutrophils are also suggested to play role in their activation and life span [117]. While neutrophils are primarily glycolytic, they rely heavily on the related PPP, the near-exclusive means of NADPH production in mammalian cells. Thus, the proposed role for HO-1 in regulating the PPP is of particular interest with regard to neutrophils [118]. HO-1 induction has been shown to reroute glucose towards PPP in cancer cells as a key modulator of PPP [118]. This pathway and the central pathway of glycolysis accommodate increased flux after neutrophil activation, providing the cell with both energy and reducing equivalents. As a relatively small proportion of the NADPH pool is needed to maintain NADPH function, upregulation of the PPP may serve to preserve the reducing capacity of the cell [119]. While it is unclear the impact this would have on neutrophils, it is likely important in a cell that exists in a delicate balance between health and harm. Ultimately, modulating HO-1 may provide a targeted approach to suppressing inflammatory neutrophil functions. Regulating their infiltration into the lung by modulating HO-1 expression, decreasing their oxidative capacity, and increasing their reductive capacity is an appealing, reversible approach to limit immunopathology in TB without deleterious effects of major immunosuppression (Figure 2). Lastly, heme degradation by HO-1 may be the predominant heme degrading mechanism but novel approaches such as targeting cytochrome P450 reductase to generate CO could be tested in heme-related tissue pathologies [120]. 

## 8. Conclusions

**Figure 3:**
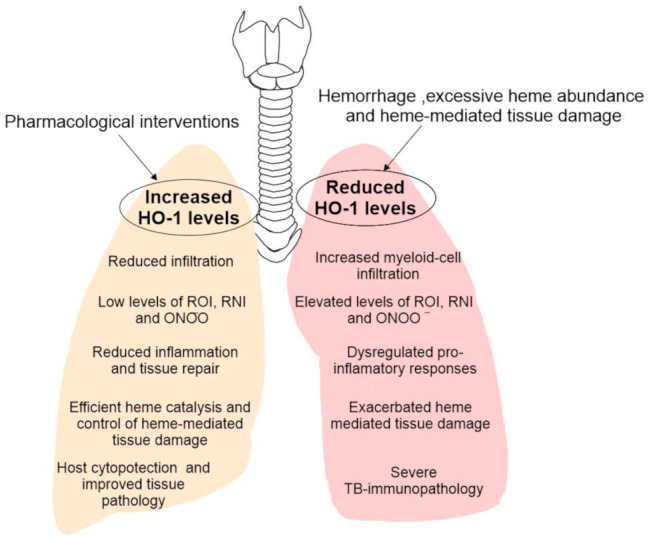
Model for the impact of HO-1 levels in the outcome of TB immunopathology. During late stages of TB disease, there is a significant hemorrhage and heme release in the surroundings of disease which causes reduction in HO-1 levels and its enzymatic activity. Heme, a very potent oxidant molecule, causes significant tissue damage. This is accompanied by uncontrolled infiltration of myeloid cells and their pro-inflammatory functions, causing significantly elevated levels of ROI, RNI and ONOO^−^ and exaggerate the TB immunopathology. Conversely, using HO-1 as HDT target and inducing its levels, especially during late stages of TB could provide host cytoprotection by improving heme catalysis, decreasing immune cell infiltration and supporting anti-inflammation and tissue repair.

Despite decades of research, we are still resolving the factors regulating the severe immunopathology that occurs during the acute and chronic stages of TB. The major pharmacological intervention against *Mtb* is often targeted towards eliminating bacteria and does little to limit host tissue damage. Additionally, the emergence of multi and extensively resistant TB, together with the increase in the cases of coinfection with HIV and other conditions such as diabetes, are compelling reasons to find new therapeutic approaches. To this end, pharmacological targeting of HO-1 or its enzymatic products could provide significant advances in improving the host tissue pathology. As observed in the recent studies, there is a wide, but distinct immune-pathological spectrum in human TB [20,28,121]. These distinct immune environments are predominantly composed of macrophages and neutrophils, which if not regulated, drives immunopathology. Therefore, to identify effective pharmacological targets to limit TB pathology, it is important to study them within the context of the TB histopathological spectrum and inflammatory status of macrophages and neutrophils within that spectrum. To this end, the diminished HO-1 levels positively correlating with significantly elevated ROS and RNS and tissue damage strongly suggest that inducing HO-1 expression may ameliorate TB immunopathology [20]. Lastly, the potential of HO-1 as a TB biomarker is also a promising area of exploration and could provide a better alternative for disease diagnosis. Taken together, pharmacologically targeting HO-1 expression provides a great potential to be tested as HDTs to limit overall disease pathology (Figure 3).
